# Prenatal Air Pollution Exposure and Early Cardiovascular Phenotypes in Young Adults

**DOI:** 10.1371/journal.pone.0150825

**Published:** 2016-03-07

**Authors:** Carrie V. Breton, Wendy J. Mack, Jin Yao, Kiros Berhane, Milena Amadeus, Fred Lurmann, Frank Gilliland, Rob McConnell, Howard N. Hodis, Nino Künzli, Ed Avol

**Affiliations:** 1 University of Southern California, Dept of Preventive Medicine, 2001 N Soto St., Los Angeles, California, 90089, United States of America; 2 University of Southern California, Atherosclerosis Research Unit, 2250 Alcazar Street, CSC 132, Los Angeles, California, 90033, United States of America; 3 Sonoma Technology Inc., 1455 N. McDowell Blvd., Suite D, Petaluma, California, 94954–6503, United States of America; 4 Swiss Tropical and Public Health Institute, Socinstr. 57, P.O. Box, 4002 Basel, Switzerland; 5 University of Basel, Petersplatz 1 CH-4003 Basel, Switzerland; Medical University Innsbruck, AUSTRIA

## Abstract

Exposure to ambient air pollutants increases risk for adverse cardiovascular health outcomes in adults. We aimed to evaluate the contribution of prenatal air pollutant exposure to cardiovascular health, which has not been thoroughly evaluated. The Testing Responses on Youth (TROY) study consists of 768 college students recruited from the University of Southern California in 2007–2009. Participants attended one study visit during which blood pressure, heart rate and carotid artery arterial stiffness (CAS) and carotid artery intima-media thickness (CIMT) were assessed. Prenatal residential addresses were geocoded and used to assign prenatal and postnatal air pollutant exposure estimates using the U.S. Environmental Protection Agency’s Air Quality System (AQS) database. The associations between CAS, CIMT and air pollutants were assessed using linear regression analysis. Prenatal PM_10_ and PM_2.5_ exposures were associated with increased CAS. For example, a 2 SD increase in prenatal PM_2.5_ was associated with CAS indices, including a 5% increase (β = 1.05, 95% CI 1.00–1.10) in carotid stiffness index beta, a 5% increase (β = 1.05, 95% CI 1.01–1.10) in Young’s elastic modulus and a 5% decrease (β = 0.95, 95% CI 0.91–0.99) in distensibility. Mutually adjusted models of pre- and postnatal PM_2.5_ further suggested the prenatal exposure was most relevant exposure period for CAS. No associations were observed for CIMT. In conclusion, prenatal exposure to elevated air pollutants may increase carotid arterial stiffness in a young adult population of college students. Efforts aimed at limiting prenatal exposures are important public health goals.

## Introduction

The negative health effects of air pollution exposure on cardiovascular risk are well documented in adults [1,2,3]. Long-term exposures have been associated with measures of atherosclerosis, including carotid intima-media thickness (CIMT) and arterial stiffness (CAS),[2,4,5,6,7,8] both of which predict future cardiovascular events in adults [9,10]. Changes in CAS, in particular, may reflect both the structural and functional health of the arterial vasculature [11]. Whether changes in CAS in children predict adult cardiovascular risk remains unknown, although recent evidence suggests blood pressure and CAS are highly related[12] and childhood blood pressure tracks closely with adult blood pressure, increasing later cardiovascular risk [13]. CAS, in fact, may be viewed as an early biomarker of endothelial function in which observed abnormalities reflect changes in the integrity of the vascular structure prior to manifestation of symptomatic cardiovascular events [14]. Plenty of evidence exists linking endothelial dysfunction to the later development of clinical vascular disease [14]. Thus, use of these surrogate vascular markers, which represent some of the best early biomarkers of adverse outcomes available in youth, may help to develop a better understanding of early vascular changes and their correlates and may also facilitate identification of children at risk for cardiovascular disease later in life [15]. Given that atherosclerosis has its origins in early life[16] an that an adverse intrauterine environment contributes to the early development of atherosclerosis, with a long latency period between exposures and adult CVD,[17] we hypothesized that exposure to air pollutants early in life may be associated with early biomarkers of cardiovascular phenotypes such as CIMT and CAS. Prenatal exposure to air pollutants may disrupt biological mechanisms that regulate fetal growth and development, which in turn may make children more susceptible to the development of cardiovascular pathologies and disease later in life. In the few studies conducted in healthy populations of children or young adults, childhood or recent exposures to air pollutants have been associated with CAS and CIMT but prenatal exposures have not been evaluated [6,18,19]. Animal models of prenatal exposure to pollutants and to tobacco smoke have demonstrated increased cardiac oxidative stress and atherogenesis in adult mice [20,21]. Pollutants such as PM_2.5_ have also been associated with systemic inflammation, oxidative stress, and endothelial injury in children and young adults [22,23,24]. To address this lack of knowledge, we investigated the association between prenatal trimester-specific and postnatal exposures to PM_10_, PM_2.5_, NO_2_ and O_3_ with CAS and CIMT in a population of University of Southern California (USC) college students.

## Methods

The Testing Responses on Youth (TROY) study consists of 768 college students recruited from USC in 2007–2009. The primary purpose of the TROY study is to assess lifetime histories of air pollution exposure in relation to early determinants of atherosclerosis. Participants were eligible for study inclusion if they were non-tobacco smokers, were born in the United States or moved to the United States within the first six months of life, and provided written informed consent to participate.

Participants attended a study visit during which CAS, CIMT, systolic and diastolic blood pressure, and heart rate were assessed by a single physician-imaging specialist from the USC Atherosclerosis Research Unit Core Imaging and Reading Center. Self-administered questionnaires were completed to gather information about health and socio-demographic characteristics as described previously [6,25]. Participants also provided a 12-hr fasting blood sample for lipid and biomarker analyses (see online supplement for further details).

The study protocol was approved by the institutional review board for human studies at the University of Southern California, and written consent was provided by the study subjects.

High-resolution B-mode ultrasound images of the right common carotid artery (CCA) were obtained with a portable Biosound MyLab 25 ultrasound system attached to a 10-MHz linear array transducer and read by a single physician-imaging specialist. Blood pressure and heart rate were measured immediately after the ultrasound examination by standard techniques after the subject was recumbent for at least ten minutes. Blood pressure was measured three times in one-minute intervals, using an OMRON blood pressure monitor with automatic cuff inflation and deflation. As previously described (Patents 2005, 2006, 2011)[6,26,27], the jugular vein and carotid artery were imaged transversely with the jugular vein stacked above the carotid artery and CIMT was measured. Media-adventitia to media-adventitia arterial dimensions were measured for calculation of the carotid arterial stiffness variables in the same arterial segment along the same 1 cm electronic ruler used to measure the CIMT using an in-house developed software package (Patents 2005, 2006, 2011) [26,27,28,29]. The lumen diameters measured during peak systole and end diastole were used to calculate three measures of arterial stiffness: distensibility, Young’s elastic modulus (YEM) and stiffness index beta (C-beta) according to standard formula (see online supplement for details) [27,30]. Duplicate scans were performed on 87 subjects and the correlation coefficients for minimum arterial diameter, maximum arterial diameter, and CIMT were 0.95, 0.95, and 0.98, respectively.

Participants completed a detailed lifetime residential history. Participant residence addresses within the U.S. were standardized and their locations were geocoded using the Tele Atlas Geocoding Service (Tele Atlas Inc., Menlo Park, California, www.na.teleatlas.com). Of the 2,598 residential locations reported, 98.3% (2,553) were U.S. residences that were successfully geocoded.

Prenatal ambient air pollution concentrations were estimated for each subject’s reported birth residence based on average monthly air pollutant exposure data and trimesters defined as follows: first trimester from 0 to 13 weeks post-conception, second trimester from 14 to 26 weeks, and third trimester from 27 to delivery. Because we previously reported an association between early childhood, elementary school and lifetime air pollution exposures with CIMT in this cohort [6], we also investigated these postnatal exposure windows with CAS. Postnatal exposure corresponding to the early childhood (0–5), elementary school years (6–12) and postnatal exposure (from birth to date of CIMT measurement) were calculated by averaging exposures across the relevant residential histories for those time periods as described previously [6]. Briefly, ambient air pollution concentrations were estimated for each subject’s residence within the U.S. from the time the subject occupied that residence to the participant’s CIMT measurement. Move-in and move-out dates were provided for each residence, and ambient air quality data was spatially interpolated to those locations for the relevant time periods using inverse distance-squared weighting (IDW2) [31,32]. The data from up to four air quality measurement stations were included in each interpolation. Due to the regional nature of O_3_, NO_2_, PM_10_, and PM_2.5_ concentrations, a maximum interpolation radius of 50 km was used for all pollutants. However, when a residence was located within 5 km of one or more stations with valid observations, the interpolation was based solely on the nearby values. A leave one out evaluation of the spatial mapping method produced r^2^ of 0.76, 0.73, 0.53, and 0.46 for monthly ozone, NO_2_, PM_2.5_, and PM_10_ concentrations using data from California (representing 85% of the population).

Air pollutant estimates were derived from the U.S. Environmental Protection Agency’s Air Quality System (AQS) database for the years 1980 through 2009. Hourly concentrations of O_3_ and NO_2_, and daily concentrations of PM_10_ and PM_2.5_ measured in all 50 states for January 1980 through 2009 were downloaded from AQS. The PM data were primarily limited to those collected with Federal Reference Method (FRM) monitors and Federal Equivalent Method (FEM). Non-FEM PM_2.5_ data were used when no FEM measurements were available. Automated quality control checks on the concentration ranges and persistence were applied to the AQS data. The AQS data were augmented in southern California with O_3_, NO_2_, PM_10_, and PM_2.5_ data from the Children’s Health Study (CHS) for 1994–2009 [33,34]. National-scale PM_10_ data were filled in using adjusted total suspended particulates (TSP) data for 1981–1987. Pre-1999 PM_2.5_ data for southern California were filled in with 1994–1998 estimated PM_2.5_ concentrations developed for the CHS.

In order to assign a postnatal exposure estimate, data were required to be 75% complete for O_3_ and NO_2_ and 12% for PM to account for the one-in-six day sampling. As a result, of 768 initial study subjects, a range of 23 up to 113 subjects could be missing trimester-specific concentrations of specific pollutants (see online supplement for more details).

Means and standard deviations of subjects’ health and anthropometric characteristics at the time of carotid ultrasound measurement as well as the distributions of prenatal and postnatal air pollutants were calculated. Air pollutants were treated as continuous variables and were scaled to a 2 standard deviation (SD) difference in level for testing associations with CAS and CIMT. The associations between CAS and CIMT and prenatal and postnatal air pollutants were assessed using linear regression analysis. Non-linear associations were evaluated using penalized splines in the GAM function of the R statistical package[35] but all associations were found to be linear. Arterial stiffness metrics were log-transformed to achieve normality. The exponentiated regression model coefficient can be interpreted as a fold-change in CAS per 2SD change in level of pollutant. Variables evaluated for confounding but not selected as confounders based on whether they changed the effect estimate of interest by greater than 10% included diastolic and systolic blood pressure, hsCRP, LDL-C, HDL-C, prenatal tobacco smoke exposure, second hand tobacco smoke exposure during childhood and homeostatic model assessment (HOMA) of insulin sensitivity and beta cell function. A final multivariate model adjusted for age, sex, race/ethnicity, maternal education, BMI, height, insulin, triglycerides, birth season and geographic region at birth for all CAS models. The final model for CIMT analysis was adjusted for age, sex, race/ethnicity, maternal education, BMI, systolic blood pressure, second hand smoke, hsCRP, LDL-C and HDL-C to be comparable to previously published results [6]. Regression procedures were conducted in SAS v9.3 (Cary, NC). [36] All statistical testing was conducted with a two-sided alpha level of 0.05.

We conducted a series of sensitivity analyses to evaluate whether exclusion of subjects by the following criteria affected our results: 1) preterm birth, 2) reported smoking of alternative tobacco products, 3) high cholesterol or high blood pressure, 4) family history of hypertension or high cholesterol, 5) family history of heart attack, heart failure or stroke, and 6) non-California born subjects; 7) poor air quality codes.

## Results

Baseline characteristics of the 768 study participants are shown in Table 1 and S1 Table in the online supplement. All participants were college students who were on average 20±1.5 years of age; the sample included more females (59%) than males (41%). Only one participant had high blood pressure (defined as > 120/80 mmHg) and family history of heart disease (5.5%) was rare in this population. C-beta, YEM, and Distensibility were log-normally distributed with geometric means (SD) of 6.2 (1.3), 2621.9 mmHg (1.4), and 30.2 x 10^−6^ x m^2^/N (1.3), respectively. These three CAS measurements were also highly correlated with one another but not with CIMT (S2 Table).

**Table 1:** Demographic characteristics of TROY participants (N = 768)*.

	N	%
Male sex	317	41.3
Race/ethnicity		
Non Hispanic White	344	44.8
Black	38	5
Asian	161	21
Hispanic White	132	17.2
Other	93	12.1
BMI^†^		
Underweight	31	4
Normal	574	74.7
Overweight	133	17.3
Obese	30	3.9
Current exposure to second-hand smoke^‡^	296	38.5
Second-hand smoke exposure during childhood	61	7.9
Ever smoked something other than cigarettes		
Yes	175	22.8
Don't know	1	0.1
Mother's Education		
High school or less	83	10.8
Some college	177	23.1
College grad/some grad school	503	65.5
Unknown	5	0.7
Family history of heart disease§		
Yes	42	5.5
Don't know	26	3.4

* TROY participants were non-smokers (of cigarettes).

^†^ Underweight was defined as BMI < 18.5, normal weight as 18.5 ≤ BMI <25, overweight as25≤ BMI <30, and obese as BMI ≥ 30.

^‡^Current second hand smoke exposure locations: Home, dormitory room, workplace, school or places other than home or school.

§History of heart attack, heart failure, or stroke.

**Fig 1:**
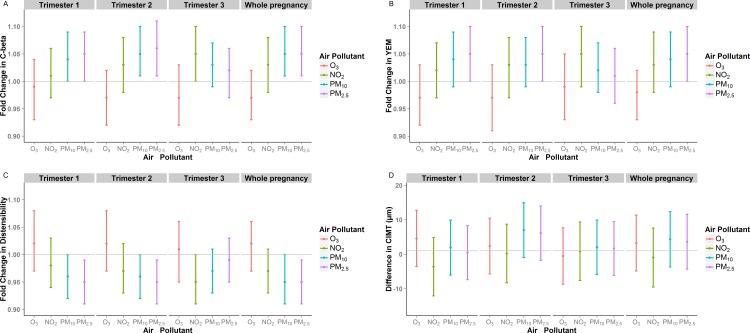
The association between prenatal air pollutant exposures and A) C-beta, B) YEM, C) Distensibility, and D) CIMT, by trimester and whole pregnancy.

Prenatal air pollutants had a range of distribution across trimesters (S3 Table). In general, NO_2_, PM_10_ and PM_2.5_ were highly correlated within trimester but less so across trimesters (S4 Table). O_3_ was not highly correlated with the other pollutants. Prenatal exposures to PM_10_, and PM_2.5_ were associated with increased CAS (Fig 1, S5 Table). For example, a 2SD higher level of PM_2.5_ during pregnancy was associated with a 5% higher C-Beta (β = 1.05, 95% CI 1.01–1.10), a 5% higher YEM (β = 1.05, 95% CI 1.01–1.10), and a 5% decrease in distensibility (β = 0.95, 95% CI 0.91–0.99). Prenatal O_3_ showed no association with CAS and prenatal NO_2_ was marginally associated. A multi-pollutant model which included both O_3_ and PM_10_ as representative of the suite of correlated pollutants did not alter interpretation of the results (Table 2). Prenatal air pollutants were not associated with CIMT (Fig 1, S5 Table).

**Table 2:** Results from a multi-pollutant model* of PM_10_ and O_3_ (N = 673).

** **	** **	**Trimester 1**			**Trimester 2**			**Trimester 3**			**Whole pregnancy**		
Outcome	Pollutant per 2SD change	fold change in outcome	95% CI		fold change in outcome	95% CI		fold change in outcome	95% CI		fold change in outcome	95% CI	
C-beta	O_3_ (ppb)	0.97	0.92	1.02	0.95	0.9	1.01	0.96	0.9	1.01	0.96	0.91	1
	PM_10_ (μ/m3)	1.06	1.01	1.11	1.07	1.02	1.12	1.04	1	1.09	1.07	1.02	1.12
YEM	O_3_ (ppb)	0.96	0.9	1.01	0.96	0.9	1.02	0.98	0.92	1.04	0.96	0.91	1.01
	PM_10_ (μ/m3)	1.06	1.01	1.11	1.05	0.99	1.1	1.04	0.99	1.09	1.06	1.01	1.11
Distensibility	O_3_ (ppb)	1.04	0.98	1.1	1.04	0.98	1.1	1.02	0.96	1.08	1.03	0.99	1.08
	PM_10_ (μ/m3)	0.94	0.9	0.99	0.95	0.91	0.99	0.96	0.92	1.01	0.94	0.9	0.99

*adjusted for sex, age, ethnicity, maternal education, BMI, height, insulin, triglycerides, birth season and geographic region

**Fig 2:**
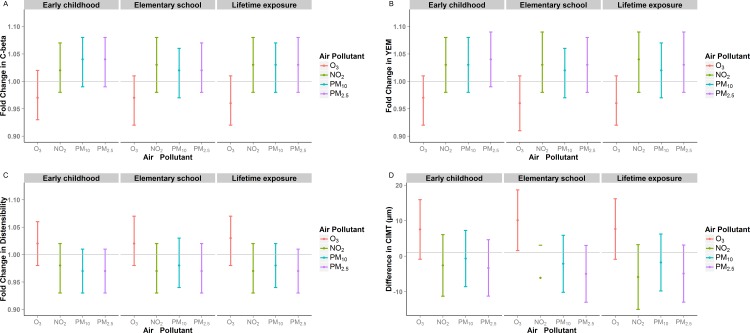
The association between postnatal air pollutant exposures and A) C-beta, B) YEM, C) Distensibility, and D) CIMT, by early childhood, elementary school and postnatal exposure.

Because we previously reported an association between early childhood, elementary school and postnatal air pollution exposures (notably O_3_) with CIMT in this cohort,[6] we also evaluated these time periods of exposure for CAS (Fig 2, S6 Table). We observed non-significant associations that were similar in magnitude to the effects observed with prenatal exposures.

We also sought to evaluate the relative contributions of prenatal and postnatal exposures on CAS and CIMT. Prenatal and postnatal NO_2_ and PM_10_ levels were highly correlated, whereas O_3_ and PM_2.5_ were moderately correlated (S7 Table). Results from models that mutually adjusted for prenatal and postnatal PM_2.5_ suggested that the effects on CAS were due to the prenatal rather than postnatal exposure (Table 3). In models that mutually adjusted for prenatal and postnatal O_3_ on CIMT, our previously reported findings of an association with postnatal O_3_ remained robust (S8 Table) whereas prenatal O_3_ had no effect on CIMT [6]. Mutually adjusted models of O_3_ on CAS showed no associations (data not shown).

**Table 3:** The association between prenatal and postnatal PM_2.5_ (μ/m^3^) exposures and CAS*(N = 724).

		Trimester 1			Trimester 2			Trimester 3			Whole pregnancy		
Outcome	Pollutant per 2SD unit change	fold change in outcome	95% CI		fold change in outcome	95% CI		fold change in outcome	95% CI		fold change in outcome	95% CI	
C-beta	Prenatal PM_2.5_	1.05	0.99	1.11	1.06	1	1.13	0.99	0.94	1.05	1.07	0.99	1.15
	Lifetime PM_2.5_	1	0.94	1.06	0.99	0.94	1.05	1.04	0.98	1.1	0.98	0.91	1.05
YEM	Prenatal PM_2.5_	1.05	0.99	1.12	1.04	0.98	1.11	0.98	0.93	1.04	1.05	0.97	1.14
	Lifetime PM_2.5_	1	0.94	1.06	1.01	0.94	1.07	1.04	0.98	1.11	0.99	0.91	1.07
Distensibility	Prenatal PM_2.5_	0.95	0.9	1	0.94	0.89	1	1.01	0.96	1.06	0.94	0.87	1
	Lifetime PM_2.5_	1	0.95	1.06	1.01	0.95	1.06	0.96	0.91	1.02	1.02	0.95	1.10

*adjusted for sex, age, ethnicity, maternal education, BMI, height, insulin, triglycerides, birth season and geographic region

Sensitivity analyses were conducted to evaluate several exclusion criteria. Removal of 68 participants who reported a family history of heart disease did not affect our results, nor did removal of 354 participants who reported a family history of hypertension or high cholesterol or removal of 40 subjects with high cholesterol or high blood pressure. Excluding the 118 participants who were born preterm or the 175 participants who reported smoking alternative tobacco products did not alter our results. Restriction of the population to participants from southern California (n = 549) on whom we had supplemental air monitoring data also did not alter our results (S9 Table). Further restriction of the population to participants who lived within 5 km from an air pollution monitor did not alter our results, though the sample size was small (S10 Table).

## Discussion

Prenatal exposure to PM_2.5_ and PM_10_ was associated with higher CAS but not CIMT in a population of college students. These results lend further evidence in support of the developmental origins of disease hypothesis for atherosclerosis [37,38,39].

Several studies in adults have demonstrated associations between long-term air pollutant exposures, particularly PM_2.5_, with CAS and CIMT [2,3,4,5,7,8,40,41], While most of these are cross-sectional in nature, longitudinal evidence is beginning to emerge [3]. PM_2.5_ is also associated with plaque burden and vascular dysfunction in murine models of atherosclerosis [3]. A few studies have demonstrated associations between air pollutants and CAS or CIMT in children or young adults [6,18,19]. Ianuzzi *et al* evaluated 52 Italian children and found that children living closer to a main road had higher CAS than those living farther away [19]. Lenters *et al* observed a 37.6% increase in augmentation index and a 4% increase in pulse wave velocity, another indicator of arterial stiffness, in response to a 25 μg/m^3^ increase in NO_2_, estimated from subjects’ residential addresses [18]. Our observed effects of a 5% increase in CAS per 2 SD (15.4 μg/m^3^ for PM_2.5_) change in pollutant level are slightly smaller in magnitude to changes in CAS observed for passive tobacco smoke exposure[30] and are comparable to an aging effect of 2.5 to 10 years during childhood [42].

In our previous report in this same study population, we observed that childhood exposure to O_3_ was associated with increased CIMT. Herein, we extend these findings to suggest that prenatal exposures ambient pollutants (PM_10_, PM_2.5_) are also important, exhibiting increases on CAS but not CIMT. One explanation for this observation may be that CAS, as a biomarker of endothelial function, reflects functionality of the arterial vasculature that may be a more sensitive marker for early subclinical cardiovascular changes in response to chronic environmental exposures whereas CIMT, a structural change, may take longer to demonstrate measurable differences.

While our observed associations were stronger and statistically significant for prenatal exposures to PM_10_ and PM_2.5_, these pollutants were correlated between prenatal and postnatal exposure periods, limiting our ability to conclude with certainty which time period confers the most risk. Nevertheless, we evaluated prenatal and postnatal PM_2.5_ and O_3_ in mutually adjusted models (pollutants with the least amount of correlation). We found that the effects of PM_2.5_ on CAS were likely due to the prenatal rather than postnatal exposure, whereas the opposite was true for O_3,_ and remained consistent with previously published results [6]. The observed effects of prenatal PM_2.5_ may occur through altered fetal growth and development. High prenatal PM exposure has been associated with lower birth weight [43] and patent ductus arteriosus [44]. PM constituents, particularly transition metals, could generate oxidative stress leading to DNA damage in the placenta, affecting the growing embryo [45]. PM may also bind receptors for placental growth factors resulting in decreased fetal–placental exchange of oxygen and nutrients, upregulate systemic pro-inflammatory mediators or alter hemodynamic responses with negative downstream consequences [45].

One of the strengths of this large study is the availability of prenatal and cumulative postnatal air pollutant exposure histories for participants. However, because we calculated air pollutant exposure estimates using existing pollutant databases acquired over twenty years prior to CAS assessment, measurement error may be of concern. To counter this, we only assigned exposure when we had relevant measurement data. Moreover, sensitivity analyses restricting the dataset to participants with only the highest quality data (i.e. in southern California for which we had additional monitoring data), as well as restricting to subjects within 5km of a monitor, yielded similar results, thereby strengthening our conclusions. A lack of monitoring data for PM_2.5_ in early years resulted in a smaller sample size for those analyses. In addition, imputation of PM_2.5_ values based on historical PM_10_/PM_2.5_ ratios may have increased measurement error. However, the pattern of results for both pollutants as well as for NO_2_ were similar, suggesting that errors specific to lack of PM_2.5_ data did not affect our results.

A general limitation to this study is the lack of information on the mothers at the time of pregnancy, including general health, habits, and occupation which could lead to unmeasured confounding. In cases where we knew maternal information, such as for preterm delivery and maternal history of cardiovascular disease or hypertension, we conducted sensitivity analyses to evaluate potential effects and found no changes to our conclusions. Traffic-related noise is another environmental stressor relevant to pregnant women and a likely contributor to vascular pathologies for which we had no data available. However, as reviewed by Tetrault *et al*, correlations between noise and traffic related pollution are rather modest [46]. Thus, our results are unlikely to be confounded by unmeasured exposure to night time noise. We studied a population of non-smoking university students who may be on average healthier and socio-economically advantaged relative to the general population. Therefore, the results of this study may not be generalizable to all individuals.

Recent guidelines for prevention of hypertension have suggested the use of vascular parameters aimed at evaluating the mechanical and functional properties of peripheral arteries in order to identify vulnerable individuals [9]. CAS is included in this list, and is considered a subclinical target in evaluating hypertensive patients [9]. Given that children rarely present with overt cardiovascular disease, use of these early vascular biomarkers in children and young adults may help to develop a better understanding of pathological vascular changes associated with air pollution exposures as well as facilitate identification of children at risk for cardiovascular disease later in life [15].

In conclusion, the atherogenic process has important determinants early in life. We present evidence that prenatal exposures to PM_2.5_ and PM_10_ are associated with CAS in a healthy population of college students. The implications of such early vascular changes with respect to adult cardiovascular disease remain unclear and require investigation. Nevertheless, regulation of air pollutants and efforts that focus on limiting prenatal and childhood exposures continue to be important public health goals to potentially reduce the atherosclerosis burden and its consequences.

## Supporting Information

S1 Final Minimal Dataset (XLSX)

S1 Methods (DOCX)

S1 Table Distribution of carotid atherosclerosis outcomes in TROY. (DOCX)

S2 Table Spearman correlation coefficients between carotid arterial stiffness measurements. (DOCX)

S3 Table Distribution of air pollutant exposures across three trimesters and the entire pregnancy. (DOCX)

S4 Table Spearman correlation within and between different pollutants across three trimester exposure windows. (DOCX)

S5 Table The association between prenatal air pollutant exposures and CAS and CIMT in young adulthood. (DOCX)

S6 Table The association between postnatal air pollutant exposures and CAS and CIMT. (DOCX)

S7 Table Spearman correlation within same pollutants between prenatal and lifetime exposures across three trimester. (DOCX)

S8 Table The association between prenatal and lifetime O_3_ (ppb) exposures and CIMT. (DOCX)

S9 Table The association between prenatal air pollutant exposures and C-beta restricted to Southern California residents. (DOCX)

S10 Table The association between prenatal air pollutant exposures and C-beta restricted to air pollution assignments within 5 km of a monitor. (DOCX)
