# Supplementary material for: Prenatal Air Pollution Exposure and Early Cardiovascular Phenotypes in Young Adults
Source: PLoS One. 2016 Mar 7;11(3):e0150825. doi: 10.1371/journal.pone.0150825 (PMC4780745; doi:10.1371/journal.pone.0150825)
Supplement: S1 Methods — (DOCX) [file pone.0150825.s002.docx]

**Supplemental Material**

**Methods**

*Health Measurements*

The lumen diameters measured during peak systole and end diastole were used to calculate three measures of arterial stiffness: distensibility, Young’s elastic modulus (YEM) and stiffness index beta (C-beta) according to standard formula. ^1, 2^ Distensibility, a measure of how much the carotid artery dilates during systole adjusted for pulse pressure (PP), was calculated using the following formula:

$$Distensibility=\frac{\left(\frac{2(D_{S}-D_{D})}{D_{D}}\right)}{PP}$$

where *D_S_* is the artery diameter at systole and *D_D_* is the artery diameter at diastole; lower values reflect a stiffer carotid artery. Distensibility was standardized to the units reported by Lage, et al. (10^-6^ x Newtons^-1^ x meters^2^).^3, 4^ The carotid stiffness index beta (C-beta) was calculated using the following formula:

*C-beta* = ln (*P_s_* / *P_d_*)

*DD*

where DD is the percentage change in carotid arterial diameter between maximum and minimum dilation ((*D_S_*  – *D_D_*)/ *D_D_* ), *P_s_* is systolic blood pressure and *P_d_* is diastolic blood pressure.

Young’s elastic modulus was calculated using the following formula:

*YEM* = PP * (*D_D_*^2^)

2*CIMT*(*D_S_* ‐ *D_D_*)

Where PP is pulse pressure, *D_S_* is the artery diameter at systole and *D_D_* is the artery diameter at diastole. For both C-beta and YEM, higher values reflect a stiffer carotid artery.

Blood pressure and heart rate were measured immediately after the ultrasound examination by standard techniques after the subject was recumbent for at least ten minutes. Blood pressure was measured three times in one-minute intervals, using an OMRON blood pressure monitor with automatic cuff inflation and deflation. Heart rate was measured using a three lead electrocardiogram as part of the Biosound MyLab 25 ultrasound system. Standing height was measured in stocking feet to the nearest centimeter using a metal measuring tape placed perpendicularly to the floor through the use of a construction-type bubble level and a measurement block to properly align head orientation. Weight was measured to the nearest pound with a medical-grade scale calibrated prior to each day’s testing using pre-determined calibration weights.

*Biologic Measurements*

Fasting plasma and serum were divided into one ml samples and stored at -80 degrees Celsius until analyzed. One ml of plasma from each subject was used to measure total cholesterol, triglycerides, and HDL cholesterol levels using an enzymatic method in conformance with the Standardization Program of the National Centers for Disease Control and Prevention. LDL-C was calculated using the Friedwald formula.^5^

One ml of serum from each subject was used to measure CRP. High-sensitivity CRP was measured by a solid-phase chemiluminescent immunometric assay using the Immulite 2000 analyzer (Siemens Medical Solutions Diagnostics, Malvern, PA). The sensitivity of the assay was 0.02 mg/dL and the inter-assay coefficient of variation was 7.0%.

*Measurement of Air Pollutants*

Participants completed a detailed lifetime residential history. Participant residence addresses within the U.S. were standardized and their locations were geocoded using the Tele Atlas Geocoding Service (Tele Atlas Inc., Menlo Park, California, www.na.teleatlas.com). Of the 2,598 residential locations reported, 98.3% (2,553) were U.S. residences that were successfully geocoded. Of the 2,553 participant residence locations: 47.8***%*** were geocoded with the highest quality match, which is specific to the centroid of the parcel or building footprint (Address Point match), and 46% were geocoded to the street segment and/or the relative position between nearest intersections. The remaining 6.2% were geocoded using Google Earth. No assignments were made for the 45 participant locations that could not be geocoded.

Postnatal exposure corresponding to the early childhood (0-5), elementary school years (6-12) and lifetime exposure (from birth to date of CIMT measurement) were calculated by averaging exposures across the relevant residential histories for those time periods as described previously.^6^

In order to assign a childhood or lifetime exposure estimate, data were required to be 75% complete for O_3_ and NO_2_ and 12% for PM to account for the one-in-six day sampling. As a result, of 768 initial study subjects, 23 were missing PM_10_ values across all trimesters, 29 were missing PM_2.5_ in trimester 1, 28 were missing PM_2.5_ in trimester 2, 25 were missing PM_2.5_ in trimester 3, 109 were missing NO_2_ in trimester 1, 113 were missing NO_2_ in trimester 2, 112 were missing NO_2_ in trimester 3, 62 were missing O_3_ for trimester 1, 61 were missing O_3_ for trimester 2, and 59 were missing O_3_ for trimester 3.

**References**

1. Selzer RH, Mack WJ, Lee PL, Kwong-Fu H, Hodis HN. Improved common carotid elasticity and intima-media thickness measurements from computer analysis of sequential ultrasound frames. Atherosclerosis 2001;**154**(1):185-93.

2. Mack WJ, Islam T, Lee Z, Selzer RH, Hodis HN. Environmental tobacco smoke and carotid arterial stiffness. Prev Med 2003;**37**(2):148-54.

3. Lage SG, Kopel L, Monachini MC, Medeiros CJ, Pileggi F, Polak JF, Creager MA. Carotid arterial compliance in patients with congestive heart failure secondary to idiopathic dilated cardiomyopathy. Am J Cardiol 1994;**74**(7):691-5.

4. Seaberg EC, Benning L, Sharrett AR, Lazar JM, Hodis HN, Mack WJ, Siedner MJ, Phair JP, Kingsley LA, Kaplan RC. Association between human immunodeficiency virus infection and stiffness of the common carotid artery. Stroke 2010;**41**(10):2163-70.

5. Hodis HN, Mack WJ, LaBree L, Mahrer PR, Sevanian A, Liu CR, Liu CH, Hwang J, Selzer RH, Azen SP. Alpha-tocopherol supplementation in healthy individuals reduces low-density lipoprotein oxidation but not atherosclerosis: the Vitamin E Atherosclerosis Prevention Study (VEAPS). Circulation 2002;**106**(12):1453-9.

6. Breton CV, Wang X, Mack WJ, Berhane K, Lopez M, Islam TS, Feng M, Lurmann F, McConnell R, Hodis HN, Kunzli N, Avol E. Childhood air pollutant exposure and carotid artery intima-media thickness in young adults. Circulation 2012;**126**(13):1614-20.

7. Peters JM, Avol E, Gauderman WJ, Linn WS, Navidi W, London SJ, Margolis H, Rappaport E, Vora H, Gong H, Jr., Thomas DC. A study of twelve Southern California communities with differing levels and types of air pollution. II. Effects on pulmonary function. Am J Respir Crit Care Med 1999;**159**(3):768-75.

8. Peters JM, Avol E, Navidi W, London SJ, Gauderman WJ, Lurmann F, Linn WS, Margolis H, Rappaport E, Gong H, Thomas DC. A study of twelve Southern California communities with differing levels and types of air pollution. I. Prevalence of respiratory morbidity. Am J Respir Crit Care Med 1999;**159**(3):760-7.
