# Supplementary material for: Prenatal Air Pollution Exposure and Early Cardiovascular Phenotypes in Young Adults
Source: PLoS One. 2016 Mar 7;11(3):e0150825. doi: 10.1371/journal.pone.0150825 (PMC4780745; doi:10.1371/journal.pone.0150825)
Supplement: S1 Table — (DOCX) [file pone.0150825.s003.docx]

**Table S1. Distribution of carotid atherosclerosis outcomes in TROY (N=768)**

|  | | | | | | |
| --- | --- | --- | --- | --- | --- | --- |
| **Variable** | **Mean** | **SD** | **Min** | **Median** | **Max** | **Q-Range** |
| CIMT (µm) | 603.4 | 54.5 | 444 | 601.75 | 767.5 | 79 |
| C-beta* | 6.2 | 1.3 | 2.2 | 6.2 | 17.9 | 2.4 |
| YEM (mmHg)* | 2621.9 | 1.4 | 785.2 | 2629.1 | 8185.6 | 1117.7 |
| Distensibility (10^-6^ x m^2^/N)* | 30.2 | 1.3 | 10.6 | 30.1 | 89.4 | 11.9 |
|  | | | | | | |

CIMT: carotid intima-media thickness, C-beta: carotid stiffness index beta, YEM: Young’s elastic modulus

*geometric mean
