# Supplementary material for: Prenatal Air Pollution Exposure and Early Cardiovascular Phenotypes in Young Adults
Source: PLoS One. 2016 Mar 7;11(3):e0150825. doi: 10.1371/journal.pone.0150825 (PMC4780745; doi:10.1371/journal.pone.0150825)
Supplement: S2 Table — (DOCX) [file pone.0150825.s004.docx]

**Table S2. Spearman correlation coefficients between carotid arterial stiffness measurements**

| **Variable** | **C-beta** | **YEM** | **N-distensibility** | **CIMT** |
| --- | --- | --- | --- | --- |
| C-beta | 1 | 0.91 | -0.96 | 0.08 |
| YEM (mmHg) |  | 1 | -0.95 | -0.11 |
| N-distensibility (10^-6^ x m^2^/N) |  |  | 1 | -0.11 |
| CIMT (µm) |  |  |  | 1 |

| ^*^All correlation coefficients had p values <0.05 |
| --- |
