# Supplementary material for: Prenatal Air Pollution Exposure and Early Cardiovascular Phenotypes in Young Adults
Source: PLoS One. 2016 Mar 7;11(3):e0150825. doi: 10.1371/journal.pone.0150825 (PMC4780745; doi:10.1371/journal.pone.0150825)
Supplement: S3 Table — (DOCX) [file pone.0150825.s005.docx]

**Table S3. Distribution of air pollutant exposures across three trimesters and the entire pregnancy**

|  | **N** | **Mean** | **SD** | **25^th^ percentile** | **50^th^ percentile** | **75^th^ percentile** |
| --- | --- | --- | --- | --- | --- | --- |
| **Trimester 1** |  |  |  |  |  |  |
| Average O_3_ (ppb) | 677 | 24 | 9.5 | 17.1 | 23.5 | 30.9 |
| Average NO_2_ (ppb) | 637 | 32.7 | 13.8 | 21.7 | 29.8 | 43.6 |
| Average PM_10_ (µ/m^3^) | 745 | 39.8 | 14.5 | 28.6 | 38.1 | 48.7 |
| Average PM_2.5_ (µ/m^3^) | 733 | 19.3 | 7.7 | 13.4 | 18.8 | 24.1 |
| **Trimester 2** |  |  |  |  |  |  |
| Average O_3_ (ppb) | 677 | 23.8 | 9.9 | 16.1 | 22.8 | 30.8 |
| Average NO_2_ (ppb) | 637 | 32.7 | 13.7 | 21.6 | 30.9 | 43.6 |
| Average PM_10_ (µ/m^3^) | 745 | 39.9 | 14.6 | 29.1 | 37.9 | 49 |
| Average PM_2.5_ (µ/m^3^) | 733 | 19.6 | 7.8 | 13.4 | 18.8 | 24.7 |
| **Trimester 3** |  |  |  |  |  |  |
| Average O_3_ (ppb) | 677 | 24.1 | 9.8 | 16.7 | 24.2 | 30.9 |
| Average NO_2_ (ppb) | 637 | 32.4 | 13.5 | 21.9 | 30 | 42 |
| Average PM_10_ (µ/m^3^) | 745 | 40.1 | 14.2 | 28.8 | 38.6 | 49.5 |
| Average PM_2.5_ (µ/m^3^) | 733 | 19.7 | 7.7 | 13.7 | 18.9 | 25.2 |
| **Whole pregnancy** |  |  |  |  |  |  |
| Average O_3_ (ppb) | 677 | 24 | 6.4 | 19.8 | 23.4 | 27.8 |
| Average NO_2_ (ppb) | 637 | 32.6 | 12.4 | 22.6 | 29.6 | 44.2 |
| Average PM_10_ (µ/m^3^) | 745 | 39.9 | 12.5 | 30.3 | 37.7 | 49.5 |
| Average PM_2.5_ (µ/m^3^) | 733 | 19.5 | 6.1 | 14.9 | 19.3 | 24.1 |
