# Supplementary material for: Prenatal Air Pollution Exposure and Early Cardiovascular Phenotypes in Young Adults
Source: PLoS One. 2016 Mar 7;11(3):e0150825. doi: 10.1371/journal.pone.0150825 (PMC4780745; doi:10.1371/journal.pone.0150825)
Supplement: S4 Table — (DOCX) [file pone.0150825.s006.docx]

**Table S4. Spearman correlation within and between different pollutants across three trimester exposure windows^*^**

|  | | | | | | | | | | | | | |
| --- | --- | --- | --- | --- | --- | --- | --- | --- | --- | --- | --- | --- | --- |
| **Exposure periods** | **Pollutant** | **Trimester 1** | | | | **Trimester 2** | | | | **Trimester 3** | | | |
|  |  | **O_3_** | **NO_2_** | **PM_10_** | **PM_2.5_** | **O_3_** | **NO_2_** | **PM_10_** | **PM_2.5_** | **O_3_** | **NO_2_** | **PM_10_** | **PM_2.5_** |
| **Trimester 1** | O_3_ | 1 | -0.15 | 0.14 | -0.01 | 0.23 |  |  |  | -0.3 |  |  |  |
|  | NO_2_ |  | 1 | 0.73 | 0.71 |  | 0.8 |  |  |  | 0.66 |  |  |
|  | PM_10_ |  |  | 1 | 0.89 |  |  | 0.72 |  |  |  | 0.56 |  |
|  | PM_2.5_ |  |  |  | 1 |  |  |  | 0.59 |  |  |  | 0.35 |
| **Trimester 2** | O_3_ |  |  |  |  | 1 | -0.24 | 0.03 | -0.14 | 0.31 |  |  |  |
|  | NO_2_ |  |  |  |  |  | 1 | 0.74 | 0.72 |  | 0.81 |  |  |
|  | PM_10_ |  |  |  |  |  |  | 1 | 0.88 |  |  | 0.7 |  |
|  | PM_2.5_ |  |  |  |  |  |  |  | 1 |  |  |  | 0.56 |
| **Trimester 3** | O_3_ |  |  |  |  |  |  |  |  | 1 | -0.19 | 0.06 | -0.12 |
|  | NO_2_ |  |  |  |  |  |  |  |  |  | 1 | 0.72 | 0.71 |
|  | PM_10_ |  |  |  |  |  |  |  |  |  |  | 1 | 0.89 |
|  | PM_2.5_ |  |  |  |  |  |  |  |  |  |  |  | 1 |
|  | | | | | | | | | | | | | |
| **Exposure periods** | **Pollutant** | **Trimester 1** | | | | **Trimester 2** | | | | **Trimester 3** | | | |
|  |  | **O_3_** | **NO_2_** | **PM_10_** | **PM_2.5_** | **O_3_** | **NO_2_** | **PM_10_** | **PM_2.5_** | **O_3_** | **NO_2_** | **PM_10_** | **PM_2.5_** |
| **Trimester 1** | O_3_ | 1 | -0.15 | 0.14 | -0.01 | 0.23 |  |  |  | -0.3 |  |  |  |
|  | NO_2_ |  | 1 | 0.73 | 0.71 |  | 0.8 |  |  |  | 0.66 |  |  |
|  | PM_10_ |  |  | 1 | 0.89 |  |  | 0.72 |  |  |  | 0.56 |  |
|  | PM_2.5_ |  |  |  | 1 |  |  |  | 0.59 |  |  |  | 0.35 |
| **Trimester 2** | O_3_ |  |  |  |  | 1 | -0.24 | 0.03 | -0.14 | 0.31 |  |  |  |
|  | NO_2_ |  |  |  |  |  | 1 | 0.74 | 0.72 |  | 0.81 |  |  |
|  | PM_10_ |  |  |  |  |  |  | 1 | 0.88 |  |  | 0.7 |  |
|  | PM_2.5_ |  |  |  |  |  |  |  | 1 |  |  |  | 0.56 |
| **Trimester 3** | O_3_ |  |  |  |  |  |  |  |  | 1 | -0.19 | 0.06 | -0.12 |
|  | NO_2_ |  |  |  |  |  |  |  |  |  | 1 | 0.72 | 0.71 |
|  | PM_10_ |  |  |  |  |  |  |  |  |  |  | 1 | 0.89 |
|  | PM_2.5_ |  |  |  |  |  |  |  |  |  |  |  | 1 |

^*^all p-values were <0.05 except O_3_ with PM_2.5_ for trimester 1, O_3_ with PM_10_ for trimester 2 and 3.
