# Supplementary material for: Prenatal Air Pollution Exposure and Early Cardiovascular Phenotypes in Young Adults
Source: PLoS One. 2016 Mar 7;11(3):e0150825. doi: 10.1371/journal.pone.0150825 (PMC4780745; doi:10.1371/journal.pone.0150825)
Supplement: S5 Table — (DOCX) [file pone.0150825.s007.docx]

| **Table S5. The association between prenatal air pollutant exposures and CAS and CIMT in young adulthood** | | | | | | | | | | | | | |
| --- | --- | --- | --- | --- | --- | --- | --- | --- | --- | --- | --- | --- | --- |
|  |  | **Trimester 1** | | | **Trimester 2** | | | **Trimester 3** | | | **Whole pregnancy** | | |
| **Outcome** | **Pollutant per 2SD change** | **β** | **95% CI** | | **β** | **95% CI** | | **β** | **95% CI** | | **β** | **95% CI** | |
| C-beta* | O_3_^‡^  (ppb) | 0.99 | 0.93 | 1.04 | 0.97 | 0.92 | 1.02 | 0.97 | 0.92 | 1.03 | 0.97 | 0.93 | 1.02 |
|  | NO_2_^§^ (ppb) | 1.01 | 0.97 | 1.06 | 1.03 | 0.98 | 1.08 | 1.05 | 1 | 1.1 | 1.03 | 0.98 | 1.08 |
|  | PM_10_^\|\|^ (µ/m^3^) | 1.04 | 1 | 1.09 | 1.05 | 1.01 | 1.1 | 1.03 | 0.99 | 1.07 | 1.05 | 1.01 | 1.10 |
|  | PM_2.5_^#^ (µ/m^3^) | 1.05 | 1 | 1.09 | 1.06 | 1.01 | 1.11 | 1.02 | 0.97 | 1.06 | 1.05 | 1.01 | 1.10 |
| YEM* | O_3_^‡^  (ppb) | 0.97 | 0.92 | 1.03 | 0.97 | 0.91 | 1.03 | 0.99 | 0.93 | 1.05 | 0.98 | 0.93 | 1.02 |
|  | NO_2_^§^ (ppb) | 1.02 | 0.97 | 1.07 | 1.03 | 0.97 | 1.08 | 1.05 | 0.99 | 1.1 | 1.03 | 0.98 | 1.09 |
|  | PM_10_^\|\|^ (µ/m^3^) | 1.04 | 0.99 | 1.09 | 1.03 | 0.99 | 1.08 | 1.02 | 0.98 | 1.07 | 1.04 | 0.99 | 1.09 |
|  | PM_2.5_^#^ (µ/m^3^) | 1.05 | 1 | 1.10 | 1.05 | 1 | 1.1 | 1.01 | 0.96 | 1.06 | 1.05 | 1 | 1.10 |
| Distensibility* | O_3_^‡^  (ppb) | 1.02 | 0.97 | 1.08 | 1.02 | 0.97 | 1.08 | 1.01 | 0.95 | 1.06 | 1.02 | 0.97 | 1.06 |
|  | NO_2_^§^ (ppb) | 0.98 | 0.94 | 1.03 | 0.97 | 0.93 | 1.02 | 0.95 | 0.91 | 1 | 0.97 | 0.93 | 1.01 |
|  | PM_10_^\|\|^ (µ/m^3^) | 0.96 | 0.92 | 1 | 0.96 | 0.92 | 1 | 0.97 | 0.93 | 1.01 | 0.95 | 0.91 | 1 |
|  | PM_2.5_^#^ (µ/m^3^) | 0.95 | 0.91 | 0.99 | 0.95 | 0.91 | 0.99 | 0.99 | 0.95 | 1.03 | 0.95 | 0.91 | 0.99 |
| CIMT^†^ | O_3_^‡^  (ppb) | 4.58 | -3.58 | 12.74 | 2.39 | -5.7 | 10.48 | -0.51 | -8.7 | 7.68 | 3.26 | -4.84 | 11.36 |
|  | NO_2_^§^ (ppb) | -3.61 | -12.09 | 4.86 | 0.23 | -8.27 | 8.74 | 0.86 | -7.62 | 9.34 | -0.96 | -9.51 | 7.59 |
|  | PM_10_^\|\|^ (µ/m^3^) | 1.94 | -6.04 | 9.92 | 7.02 | -0.93 | 14.96 | 2.04 | -5.84 | 9.91 | 4.34 | -3.69 | 12.38 |
|  | PM_2.5_^#^ (µ/m^3^) | 0.49 | -7.35 | 8.33 | 6.12 | -1.77 | 14.01 | 1.67 | -6.11 | 9.46 | 3.62 | -4.32 | 11.57 |

^*^β is the fold change in effect estimate, adjusted for sex, age, ethnicity, maternal education, BMI, height,insulin, triglycerides, birth season and geographic region.

^†^β is the difference in CIMT (µm), adjusted for ethnicity, maternal education, sex, age at IMT, BMI, systolic blood pressure, current second-hand smoke, child second-hand smoke, hscrp, HDL, and LDL.

^‡^N=677, ^§^N=637, ^||^N=745, ^#^N=733
