# Supplementary material for: Prenatal Air Pollution Exposure and Early Cardiovascular Phenotypes in Young Adults
Source: PLoS One. 2016 Mar 7;11(3):e0150825. doi: 10.1371/journal.pone.0150825 (PMC4780745; doi:10.1371/journal.pone.0150825)
Supplement: S6 Table — (DOCX) [file pone.0150825.s008.docx]

**Table S6. The association between early childhood air pollutant exposures and CAS and CIMT in young adulthood**

|  |  | **Early childhood (ages 0-5)** | | | **Elementary school exposure (ages 5-12)** | | | **Postnatal exposure** | | |
| --- | --- | --- | --- | --- | --- | --- | --- | --- | --- | --- |
| **Outcome** | **Pollutant per 2SD change** | **β** | **95% CI** | | **β** | **95% CI** | | **β** | **95% CI** | |
| C-beta* | O_3_^†^  (ppb) | 0.97 | 0.93 | 1.02 | 0.97 | 0.92 | 1.01 | 0.96 | 0.92 | 1.01 |
|  | NO_2_^‡^ (ppb) | 1.02 | 0.98 | 1.07 | 1.03 | 0.98 | 1.08 | 1.03 | 0.98 | 1.08 |
|  | PM_10_^§^ (µ/m^3^) | 1.04 | 0.99 | 1.08 | 1.02 | 0.97 | 1.06 | 1.03 | 0.98 | 1.07 |
|  | PM_2.5_^\|\|^ (µ/m^3^) | 1.04 | 0.99 | 1.08 | 1.02 | 0.98 | 1.07 | 1.03 | 0.98 | 1.08 |
| YEM* | O_3_^†^  (ppb) | 0.97 | 0.92 | 1.01 | 0.96 | 0.91 | 1.01 | 0.96 | 0.92 | 1.01 |
|  | NO_2_^‡^ (ppb) | 1.03 | 0.98 | 1.08 | 1.03 | 0.98 | 1.09 | 1.04 | 0.98 | 1.09 |
|  | PM_10_^§^ (µ/m^3^) | 1.03 | 0.98 | 1.08 | 1.02 | 0.97 | 1.06 | 1.02 | 0.97 | 1.07 |
|  | PM_2.5_^\|\|^ (µ/m^3^) | 1.04 | 0.99 | 1.09 | 1.03 | 0.98 | 1.08 | 1.03 | 0.98 | 1.09 |
| Distensibility* | O_3_^†^  (ppb) | 1.02 | 0.98 | 1.06 | 1.02 | 0.98 | 1.07 | 1.03 | 0.98 | 1.07 |
|  | NO_2_^‡^ (ppb) | 0.98 | 0.93 | 1.02 | 0.97 | 0.93 | 1.02 | 0.97 | 0.93 | 1.02 |
|  | PM_10_^§^ (µ/m^3^) | 0.97 | 0.93 | 1.01 | 0.98 | 0.94 | 1.03 | 0.98 | 0.94 | 1.02 |
|  | PM_2.5_^\|\|^ (µ/m^3^) | 0.97 | 0.93 | 1.01 | 0.97 | 0.93 | 1.02 | 0.97 | 0.93 | 1.01 |

^*^β is the fold change in effect estimate, adjusted for sex, age, ethnicity, maternal education, BMI, height,insulin, triglycerides, birth season and geographic region.

^†^N=660, ^‡^N=640, ^§^N=754, ^||^N=728
