# Supplementary material for: Prenatal Air Pollution Exposure and Early Cardiovascular Phenotypes in Young Adults
Source: PLoS One. 2016 Mar 7;11(3):e0150825. doi: 10.1371/journal.pone.0150825 (PMC4780745; doi:10.1371/journal.pone.0150825)
Supplement: S7 Table — (DOCX) [file pone.0150825.s009.docx]

**Table S7. Spearman correlation within same pollutants between prenatal and postnatal exposures across three trimester^*^**

| **Exposure periods** | **Pollutant** | **Trimester 1** | | | | **Trimester 2** | | | | **Trimester 3** | | | |
| --- | --- | --- | --- | --- | --- | --- | --- | --- | --- | --- | --- | --- | --- |
|  |  | **O_3_** | **NO_2_** | **PM_10_** | **PM_2.5_** | **O_3_** | **NO_2_** | **PM_10_** | **PM_2.5_** | **O_3_** | **NO_2_** | **PM_10_** | **PM_2.5_** |
| **Postnatal** | O_3_ | 0.42 |  |  |  | 0.39 |  |  |  | 0.45 |  |  |  |
|  | NO_2_ |  | 0.83 |  |  |  | 0.81 |  |  |  | 0.81 |  |  |
|  | PM_10_ |  |  | 0.73 |  |  |  | 0.72 |  |  |  | 0.72 |  |
|  | PM_2.5_ |  |  |  | 0.67 |  |  |  | 0.66 |  |  |  | 0.66 |

^*^all p-values were <0.05.
