# Supplementary material for: Prenatal Air Pollution Exposure and Early Cardiovascular Phenotypes in Young Adults
Source: PLoS One. 2016 Mar 7;11(3):e0150825. doi: 10.1371/journal.pone.0150825 (PMC4780745; doi:10.1371/journal.pone.0150825)
Supplement: S8 Table — (DOCX) [file pone.0150825.s010.docx]

**Table S8. The association between prenatal and postnatal O_3_ (ppb) exposures and CIMT*(N=647)**

|  | **Trimester 1**^†^ | | | **Trimester 2**^‡^ | | | **Trimester 3**^§^ | | | **Whole pregnancy**^\|\|^ | | |
| --- | --- | --- | --- | --- | --- | --- | --- | --- | --- | --- | --- | --- |
| **Pollutant per 2SD unit change** | **Difference in IMT (µm)** | **95% CI** | | **Difference in IMT (µm)** | **95% CI** | | **Difference in IMT (µm)** | **95% CI** | | **Difference in IMT (µm)** | **95% CI** | |
| Prenatal O_3_ | 1.34 | -7.79 | 10.47 | -1.18 | -10.21 | 7.84 | -5.52 | -14.82 | 3.79 | -3.84 | -14.69 | 7.01 |
| Postnatal O_3_ | 8.37 | -1.31 | 18.06 | 9.5 | -0.21 | 19.21 | 11.51 | 1.71 | 21.31 | 11.65 | 0.03 | 23.27 |

*adjusted for ethnicity, sex, age at IMT, maternal education, BMI, systolic blood pressure, current second-hand smoke, child second-hand smoke, hscrp, HDL, and LDL.
