# Supplementary material for: Prenatal Air Pollution Exposure and Early Cardiovascular Phenotypes in Young Adults
Source: PLoS One. 2016 Mar 7;11(3):e0150825. doi: 10.1371/journal.pone.0150825 (PMC4780745; doi:10.1371/journal.pone.0150825)
Supplement: S9 Table — (DOCX) [file pone.0150825.s011.docx]

**Table S9. The association between prenatal air pollutant exposures and C-beta restricted to Southern California residents (N=549)^*^**

|  | **Trimester 1** | | | **Trimester 2** | | | **Trimester 3** | | | **Whole pregnancy** | | |
| --- | --- | --- | --- | --- | --- | --- | --- | --- | --- | --- | --- | --- |
| **Pollutant per 2SD unit change** | **fold change in C-beta** | **95% CI** | | **fold change in C-beta** | **95% CI** | | **fold change in C-beta** | **95% CI** | | **fold change in C-beta** | **95% CI** | |
| O_3_^†^  (ppb) | 1 | 0.94 | 1.07 | 0.97 | 0.91 | 1.03 | 0.97 | 0.91 | 1.03 | 0.98 | 0.93 | 1.03 |
| NO_2_^‡^ (ppb) | 1 | 0.95 | 1.05 | 1.01 | 0.96 | 1.07 | 1.04 | 0.98 | 1.09 | 1.02 | 0.97 | 1.07 |
| PM_10_^§^ (µ/m^3^) | 1.05 | 1 | 1.10 | 1.07 | 1.01 | 1.12 | 1.03 | 0.98 | 1.09 | 1.06 | 1.01 | 1.12 |
| PM_2.5_^\|\|^ (µ/m^3^) | 1.04 | 0.99 | 1.09 | 1.07 | 1.02 | 1.13 | 1.01 | 0.96 | 1.06 | 1.05 | 1 | 1.11 |

*adjusted for sex, age, ethnicity, maternal education, BMI, height, insulin, triglycerides, birth season and geographic region

^†^N=527, ^‡^N=514, ^§^N=541, ^||^N=537
