# Supplementary material for: Prenatal Air Pollution Exposure and Early Cardiovascular Phenotypes in Young Adults
Source: PLoS One. 2016 Mar 7;11(3):e0150825. doi: 10.1371/journal.pone.0150825 (PMC4780745; doi:10.1371/journal.pone.0150825)
Supplement: S10 Table — (DOCX) [file pone.0150825.s012.docx]

**Table S10. The association between prenatal air pollutant exposures and C-beta restricted to air pollution assignments within 5 km of a monitor ***

|  | **Trimester 1** | | | **Trimester 2** | | | **Trimester 3** | | | **Whole pregnancy** | | |
| --- | --- | --- | --- | --- | --- | --- | --- | --- | --- | --- | --- | --- |
| **Pollutant per 2SD change** | **β** | **95% CI** | | **β** | **95% CI** | | **β** | **95% CI** | | **β** | **95% CI** | |
| O_3_^†^  (ppb) | 0.91 | 0.8 | 1.03 | 0.85 | 0.74 | 0.98 | 0.87 | 0.75 | 1 | 0.88 | 0.79 | 0.98 |
| NO_2_^‡^ (ppb) | 0.95 | 0.83 | 1.08 | 1.02 | 0.87 | 1.19 | 1.01 | 0.86 | 1.19 | 0.98 | 0.85 | 1.14 |
| PM_10_^§^ (µ/m^3^) | 0.99 | 0.88 | 1.1 | 1.04 | 0.92 | 1.19 | 1.04 | 0.92 | 1.17 | 1.02 | 0.90 | 1.16 |
| PM_2.5_^\|\|^ (µ/m^3^) | 0.99 | 0.89 | 1.1 | 1.05 | 0.93 | 1.18 | 1.03 | 0.91 | 1.17 | 1.02 | 0.91 | 1.15 |
|  | |  |  |  |  |  |  |  |  |  |  |  |

*adjusted for sex, age, ethnicity, maternal education, BMI, height, insulin, triglycerides, birth season and geographic region

^†^N=143, ^‡^N=95, ^§^N=116, ^||^N=119
